# Animal Models to Study Hepatitis C Virus Infection

**DOI:** 10.3389/fimmu.2018.01032

**Published:** 2018-05-14

**Authors:** Rani Burm, Laura Collignon, Ahmed Atef Mesalam, Philip Meuleman

**Affiliations:** ^1^Laboratory of Liver Infectious Diseases, Department of Clinical Chemistry, Microbiology and Immunology, Faculty of Medicine and Health Sciences, Ghent University, Gent, Belgium; ^2^Therapeutic Chemistry Department, National Research Centre (NRC), Cairo, Egypt

**Keywords:** hepatitis C virus, animal models, humanized mice, homologs, vaccine, antiviral therapy

## Abstract

With more than 71 million chronically infected people, the hepatitis C virus (HCV) is a major global health concern. Although new direct acting antivirals have significantly improved the rate of HCV cure, high therapy cost, potential emergence of drug-resistant viral variants, and unavailability of a protective vaccine represent challenges for complete HCV eradication. Relevant animal models are required, and additional development remains necessary, to effectively study HCV biology, virus–host interactions and for the evaluation of new antiviral approaches and prophylactic vaccines. The chimpanzee, the only non-human primate susceptible to experimental HCV infection, has been used extensively to study HCV infection, particularly to analyze the innate and adaptive immune response upon infection. However, financial, practical, and especially ethical constraints have urged the exploration of alternative small animal models. These include different types of transgenic mice, immunodeficient mice of which the liver is engrafted with human hepatocytes (humanized mice) and, more recently, immunocompetent rodents that are susceptible to infection with viruses that are closely related to HCV. In this review, we provide an overview of the currently available animal models that have proven valuable for the study of HCV, and discuss their main benefits and weaknesses.

## Introduction

The worldwide prevalence of hepatitis C virus (HCV) infection is 3% with an estimated 71 million people who are persistently infected. The severity of HCV infection ranges from mild symptoms to serious illness with chronic hepatitis. Chronic infection may lead to liver cirrhosis and eventually hepatocellular carcinoma (HCC) (1). In recent years, new direct acting antivirals (DAAs) have first supplemented the treatment combination of ribavirin and pegylated interferon alpha (IFNα), reaching cure rates of up to 90% in genotype 1 infected patients. The latest DAA combinations are even more effective and do not require additional ribavirin or interferon administration. Despite these recent advances, significant concerns remain about drug resistance, high cost, and worldwide accessibility of these new antivirals. Besides, DAAs do not necessarily ameliorate the long-term effects of chronic infection and predisposition for liver disease (2). In addition, since therapy-induced HCV clearance does not provide immunity to a new infection, an effective preventive vaccine remains an important need (3).

The first accessible system to study HCV replication in cell culture was the sub-genomic replicon system (4). This approach allows efficient viral replication in human hepatoma (Huh7) cells, transfected with sub-genomes that contain a selectable marker linked to the non-structural region (NS2-NS5B) of HCV (4, 5). Using this system, HCV RNA replication and cellular immunity (6, 7) can be studied and novel antiviral compounds (8) can be evaluated. Important to note is that no infectious viral particles are produced using this sub-genomic replicon system. In parallel, *in vitro* systems for the study of viral entry were developed. Virus-like particles, produced in a baculovirus system and containing the structural proteins core, E1 and E2, resemble HCV virions and are capable of inducing humoral immune responses against HCV (9). However, these particles are not secreted and have no infectious potential. The first infectious systems consisted of pseudotyped vesicular stomatitis virus or influenza virus containing chimeric E1 and/or E2 glycoproteins (10–13). However, due to modifications that allow assembly at the cell surface, the conformation and functions of the E1/E2 complexes are disturbed (13). The development of infectious HCV pseudo-particles (HCVpp), which consist of defective retroviral particles expressing HCV E1 and E2 glycoproteins on their surface, represented a major breakthrough for investigating the HCV entry process (14–16). More specifically, the role of putative HCV (co-) receptors, the host range, and the E1 and E2 glycoproteins can be examined. This system also allows screening of potential entry inhibitors. In this way, the HCVpp are shown to be hepatotropic and can specifically be neutralized by anti-E2 monoclonal antibodies and HCV-infected patient sera (15). Further steps in the HCV life cycle are not supported by HCVpp and can, therefore, not be explored using HCVpp (15). In 2005, transfection of *in vitro* transcribed full-length genotype 2a HCV (JFH1) isolate and chimeric derivatives thereof into Huh7 cells was described, showing RNA replication and secretion of infectious viral particles (17–20). In contrast to the HCVpp system, this cell culture-derived HCV (HCVcc) system allows the study of all aspects of the viral life cycle and still plays a major role in the identification and evaluation of novel antivirals (19, 20).

**Figure 1:**
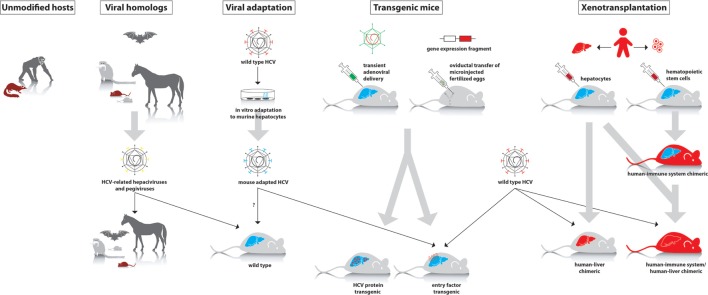
Different approaches to study hepatitis C virus (HCV) in animal models. First panel: animal species that can be experimentally infected with wild-type HCV (chimpanzee and tree shrew). Second panel: hepaciviruses and pegiviruses that infect animal species such as wild mice, rats, tamarins, bats, and horses. These viruses can be studied in their natural host, where they cause a HCV-like infection. Third panel: *in vitro* adaptation of HCV to mouse hepatocytes may allow the isolation of viral variants that can establish an infection in wild-type mice. Fourth panel: transient or stable expression of human factors that are essential to support infection of wild-type HCV or transgenic expression of viral proteins. Fifth panel: in xenotransplantation models, either the liver alone or both the liver and immune system are humanized.

Cell culture systems are very useful for initial studies of different aspects of HCV. However, culture conditions are artificial; hence, *in vivo* studies are required to more closely mimic the natural situation. Due to the narrow tropism of HCV, *in vivo* studies were long restricted to chimpanzees. Over the years, other animal species have been evaluated for their susceptibility to HCV infection, although most of them seemed resistant. Therefore, several modified models have been developed in recent years, which allow either complete or partial study of HCV infection. In this review, we provide an overview of currently existing *in vivo* models for HCV infection. We will also discuss their applicability, major advantages, and limitations (Table 1; Figure 1).

**Table 1:** Characteristics of hepatitis C virus (HCV) animal models and HCV homologs.

Animal model	Complete viral life cycle	Viremia	Liver disease	DAA testing	Passive immunization	Vaccine development	Availability	Reference
**Non-rodent models**								
Chimpanzee	Yes	High	Acute, chronic^a^	Yes	Yes	Yes	Very low	(37, 39, 45)
Tree shrew	Yes	Low	Fibrosis, cirrhosis	No	Yes	No	Low	(54, 55)
Zebrafish	Replication	Not relevant	Virus–host interaction	Yes	No	No	High	(56)

**Viral protein transgenic mouse models**								
Inducible transgene expression	Not relevant	Not relevant	Virus–host interaction	Not relevant	Not relevant	No	High	(58, 67, 68)
Full HCV genome	Not relevant	Not relevant	Fibrosis, HCC	Not relevant	Not relevant	No	High	(70)

**Immunocompromised human liver xenograft mouse models**								
Trimera mouse	Yes	Low	No	Yes	Yes	No	Low	(78, 80)
Alb-uPA-SCID mouse with humanized liver	Yes	High	No	Yes	Yes	No	Low	(79, 83, 88, 90–92)
FRG mouse	Yes	High	No	Yes	Yes	No	Low	(96, 130, 133, 134, 136, 137)

**Immunocompetent xenograft mouse models**								
Tolerized rat	Yes	Very low	No	Yes	Yes	Yes	Very low	(139, 141)
AFC8-hu HSC/Hep mouse	Yes	Only in liver	Inflammation, fibrosis	No	No	Yes	Very low	(142)
HIL mouse	Yes	Very low	Inflammation, fibrosis	No	No	Yes	Very low	(155, 156)

**Viral adaptation**								
	Entry	No	No	No	Yes	No	High	(157, 158)

**Genetically humanized mouse models**								
Rosa26-Fluc mouse	Yes	Persistent viremia	No	Yes	Yes	Yes	High	(159, 160)
ICR-C/OTg mouse	Yes	Persistent viremia	Fibrosis	Yes	Yes	Yes	High	(161)

**HCV homologs in natural host**								
GB-virus	Yes	Acute	No	Yes^b^	Yes^b^	Yes^b^	Low	(165)
NPHV in horses	Yes	Persistent viremia, acute	Inflammation	Yes^b^	Yes^b^	Yes^b^	Low	(168, 169)
NrHV in rats	Yes	Acute, chronic	Inflammation	Yes^b^	Yes^b^	Yes^b^	High	(172–174)

*DAA, direct-acting antiviral; HCC, hepatocellular carcinoma; uPa, urokinase-type plasminogen activator; HSC, hematopoietic stem cell; HIL, human immune system and liver; NPHV, non-primate hepacivirus; NrHV, Norway rat hepacivirus*.

*^a^Chimpanzees are not necessarily good models of chronic liver disease. They do not develop fibrosis or HCC*.

*^b^These models can be used to evaluate the efficacy of DAAs and vaccine candidates against the homolog virus, not HCV itself*.

## Host System Requirements for HCV Replication

As with any experimental system for human disease, a model for HCV infection should mimic as many, if not all, relevant clinical features as observed in human patients. Desirably, the model should be susceptible to all HCV genotypes with resulting persistent viremia in the majority of exposed animals. The ideal model should also be fully immunocompetent in order to study protective immunity, persistence, and immune-mediated pathogenesis. From a practical point of view, the animal model for HCV infection should be cheap, highly reproducible, easy to propagate and high in throughput (21). Finally, the ethical impact should be as minimal as possible. Up to this day, no such model exists.

Since the number of unmodified hosts perceptive to HCV infection is limited, extensive research is performed to create a suitable model by modifying existing models. From all animal models used in research, rodents are currently the most popular species for genetic modifications and are therefore highly explored, also in the field of HCV research. Genetic manipulation of the host can be applied to knock down certain host factors that interfere with viral replication or on the other hand, to complement the host with human factors that are essential for this process. The propagation of HCV in rodent cells is inefficient, presumably due to genetic incompatibility of rodent cofactors and/or due to suppression of HCV replication by rodent innate immune defenses. Thus, engineering mice expressing the relevant human genes and/or with deleted mouse restriction factors may permit HCV propagation (22).

A large number of human factors have been determined to be involved in the uptake of HCV into human hepatocytes: glycosaminoglycans (23), low density lipoprotein receptor (24), CD81 (25), scavenger receptor class B type 1 (SR-BI) (26), tight junction proteins claudin-1 (CLDN1) (27) and occludin (OCLN) (28, 29), the receptor tyrosine kinases epidermal growth factor receptor and ephrin receptor A2 (30), the cholesterol transporter Niemann-Pick C1-like 1 (31), transferrin receptor 1 (32), cell death-inducing DFFA-like effector b (33), and E-cadherin (34). The entry of HCV into primary hepatocytes is mediated by CD81, OCLN, CLDN1, and SR-BI. To our current knowledge, CD81 and OCLN comprise the minimal human factors required for HCV uptake by rodent cells (35). However, these animals do not sustain viral replication and chronic infection. Finally, it is still not entirely clear which host factors should be humanized, because there is little knowledge about the specific host factors that cause inhibition of HCV RNA replication or host factors that determine species tropism.

## Non-Rodent Models

The chimpanzee (*Pan troglodytes*) played an important role in the discovery of HCV. In fact, the viral genome of HCV was cloned from a chimpanzee that was experimentally infected with non-A, non-B hepatitis (36). For a long time, the chimpanzee was the only available model to study HCV, and their use has greatly advanced our knowledge on this virus. Humans and chimpanzees share more than 98% of their genome sequence. Despite this high genomic homology, there are some clear differences between the two which makes that the disease pattern and outcome in chimpanzees does not necessarily reflect that in humans. Whereas only a minority of humans spontaneously clear an acute infection (15%), few chimpanzees evolve to chronicity (30–40%) (37), and to date, no fibrosis and only one HCC case has been observed in this model (38). Nevertheless, the chimpanzee proved very valuable for the study of the molecular, immunological, and clinical aspects of HCV infection. Furthermore, while it is very difficult to study the acute phase of HCV infection in humans because specific symptoms are usually absent during that phase, experimental infection of chimpanzees allows close monitoring of viral kinetics, host immune response, disease manifestation, and outcome in a highly controlled manner (39–43). Immunological studies in chimpanzees have also led to the development and evaluation of several candidate vaccines (44, 45). Moreover, in the context of antiviral efficacy studies, they have been successfully used to track resistance associated with the use of entry (46), protease, NS5A (47), and polymerase (48, 49) inhibitors and combinations thereof (50).

The chimpanzee model fulfills many of the requirements for a good animal model. However, limited availability and ethical and financial constraints associated with these studies are major drawbacks. Recently, the National Institute of Health of the United States Department of Health and Human Services decided to effectively end its support for invasive research on chimpanzees. Other primates have been tested for their susceptibility to HCV infection, with little success. Although HCV can infect induced pluripotent stem cells derived from hepatocyte-like cells from pigtail macaques (51, 52), HCV does not seem to be able to establish persistent infection in non-human primates except for chimpanzees (53).

In addition, several other non-primate species have been tested for their susceptibility to HCV. The tree shrew (*Tupaia belangeri*) is for example a non-rodent squirrel-like mammal that is permissive for persistent low-level HCV viremia, including HCV-related liver disorders (54, 55). Still, limited availability and incompatibility of the *Tupaia* host environment with robust HCV replication limits the use of this animal for the study of HCV pathogenesis and vaccine development.

Recently, Ding et al. (56) developed a zebrafish model for sub-genomic HCV replication. The zebrafish is often used as a model organism for human diseases, including liver disease (57). The sub-replicon is created using two vectors: one containing HCV NS5B and the other containing the minus strand of HCV 5′UTR, core, and 3′UTR, under the control of the mouse hepatocyte nuclear factor 4 promoter. These vectors are then co-injected into zebrafish zygotes. The sub-replicon is able to replicate in the liver and causes alterations in the expression of certain genes, which is similar to HCV infection in human liver cells. Administration of ribavirin and oxymatrine significantly inhibits the replication of the HCV sub-replicon in the larvae (56). To conclude, the zebrafish is small, easy to handle experimentally, and useful for investigating mechanisms of HCV replication and liver pathology *in vivo*. Also, this model may aid in drug evaluation studies and thus the discovery of new anti-HCV drugs.

## Viral Protein Transgenic Mice

Mice that transgenically express viral proteins have been created to study the *in vivo* interactions between these viral proteins and the host cell. Transgenic mice, containing the genetic code for HCV structural proteins E1, E2 or core (or combinations thereof); or the non-structural NS3/4A protein, show conflicting results in the development of liver pathologies. Some reports do not show any evidence of hepatocellular damage (58–61), while other groups describe progressive hepatic steatosis and HCC (62–65). These discrepancies may be explained by the relationship between inflammation-associated hepatocarcinogenesis and the host genetic background (66). A major drawback of these HCV-transgenic mice is that the transgene integrates randomly and in high copy numbers. Consequently, the viral proteins are highly overexpressed, often in an uncontrolled manner. Certain aspects of the HCV-transgenic mouse phenotype may be attributed to the artificial overexpression and/or interference with the regulation of genes located near the integration site. If the expression of viral proteins can be controlled and fine-tuned, the limitations of these models may be overcome. The Cre/Lox system (67) or hydrodynamic injection (68) allows inducible expression of the transgene. Using the murine major urinary protein (MUP)-promoter, the expression can be delayed (58). The immune system of this model closely resembles that of a chronically infected patient. Hence, it allows the evaluation of potential therapeutic vaccine strategies (69). Lerat et al. (70) created a transgenic FL-N/35 mouse model expressing the full HCV genome at levels corresponding to natural human infection (70, 71). The FL-N/35 mouse model is certainly the most relevant transgenic mouse model available at this time, especially for investigating hepatic steatosis, fibrosis, and HCC.

## Immunocompromised (Human) Liver Xenograft Mouse Models

Because mice are naturally not susceptible to HCV infection, an interesting approach to overcome the species barrier is by humanizing the liver *via* transplantation of primary human hepatocytes. In this way, mice can not only be infected with HCV but also with other human hepatotropic pathogens. However, if immunocompetent rodents are transplanted with xenogeneic hepatocytes, rejection of donor cells by the host cellular immune system is observed (72–74). In order to prevent this rejection, mice need to be immunocompromised. In addition, recipients must suffer from some type of liver disease to ablate murine hepatocytes and to allow proliferation of donor hepatocytes in the mouse liver parenchyma. This liver injury can be generated in three ways: chemically, surgically, or genetically (75). Several humanized mouse models have been developed and explored for HCV infection during the past 20 years.

### The Trimera Mouse Model

The Trimera mouse was the first chimeric model and is composed of three genetically disparate sources of tissue (i.e., recipient mouse, bone marrow donor mouse, and human liver tissue), hence its name (76). After the recipient mouse is preconditioned by lethal total body irradiation, it is radioprotected by immediate injection of bone marrow cells from an immunodeficient SCID mouse (76). Then, human liver fragments, infected *ex vivo* with hepatotropic virus, are transplanted in ectopic sites of the recipient mouse such as the ear pinna or under the kidney capsule (76–78). Using this method, Ilan et al. (77, 78) were able to generate mice that can be infected with HBV and HCV. Higher serum HCV loads are obtained when pre-infected liver fragments from HCV-positive patients are employed compared to *ex vivo* infected liver fragments (78). HCV viremia persists for approximately 1 month and declines thereafter as a result of fibrosis and necrosis of the human graft (78). These observed histological abnormalities of the transplant can be attributed to their transplantation at an extrahepatic location (79). Also, *de novo* infection of Trimera mice, transplanted with healthy liver grafts, has so far not been achieved. This means that viral entry or neutralization studies cannot be performed using this model (79). Nevertheless, the 1-month time window may be sufficient for the evaluation of certain anti-HCV therapeutics or HCV vaccines (78). In fact, an HCV internal ribosomal entry site inhibitor was successfully tested in the Trimera mouse model (78, 80).

### The Alb-uPA-SCID Mouse Model With Humanized Liver and Variants

The Alb-uPA mouse model was initially designed to study the pathophysiology of plasminogen hyperactivation and to evaluate new therapy regimens for bleeding disorders (81). These transgenic mice carry a tandem repeat of four murine urokinase-type plasminogen activator (uPA) genes under the control of a mouse albumin (Alb) promoter/enhancer (Alb-uPA mice) (81, 82). The hepatic uPA transgene overexpression results in elevated uPA plasma levels, but also leads to accelerated hepatocyte death, hypofibrinogenemia, and serious hemorrhagic events such as intra-abdominal and intestinal bleedings in neonatal transgenic mice (81–83). However, the high uPA concentration gradually returns to normal levels by the age of 2 months (82). This is probably due to somatic deletions of (parts of) the transgene construct within hepatocytes (82). Consequently, these transgene-deficient cells can selectively proliferate and regenerate the diseased liver tissue (82). On the other hand, when newborn Alb-uPA mice are transplanted with healthy donor hepatocytes, their functional liver deficit is also restored by the transplanted cells that repopulate the diseased liver (82, 84). In order to prevent rejection of hepatocyte transplants of xenogeneic origin, Alb-uPA mice should be backcrossed to an immunotolerant genetic background (84).

Mouse, rat, and woodchuck hepatocytes can be successfully transplanted into immunodeficient Alb-uPA mice using intrasplenic injection (84–86). Mouse livers are chimerically composed of both donor-derived and host-derived cells, the latter having a survival advantage by deletion of (parts of) the transgene (84–86). This transgene inactivation occurs less frequently in homozygous uPA animals compared to their hemizygous counterparts, because in the former two transgene arrays must be inactivated which is less likely to occur (82, 86). Accordingly, liver chimerism can be sustained for a much longer period and at higher levels in homozygous mice (86). Up to 90% of the liver may be reconstituted with donor hepatocytes and initially these cells appear to grow in a nodular fashion (84, 85).

By extension, this model is suitable for evaluating the susceptibility of donor hepatocytes to liver infections with a specific tropism for the donor species. Petersen et al. (85) were able to detect persistent non-cytotoxic woodchuck hepatitis virus infection in chimeric livers of uPA/recombination activating gene 2 (RAG2) mice transplanted with woodchuck hepatocytes (85). Accordingly, the same group (87) was able to transplant adult human hepatocytes early after birth in immunotolerant uPA/RAG2 mice. Up to 15% of the livers consist of human hepatocytes and homozygosity of the Alb-uPA transgene is also required to ensure sustained human engraftment (83, 87). The human hepatocytes seem to repopulate the liver in a well-organized fashion with preservation of normal cell function and pharmacological responses (88, 89). In addition, human albumin, which indicates the functionality of the chimeric liver, is detected in plasma for at least 2 months after transplantation (87). Besides mature human hepatocytes, also hepatic progenitor cells are observed in these livers (90). Better humanization is obtained using commercially available, cryopreserved human hepatocytes (91). Remarkably, after inoculation with human HBV infectious serum, productive infection is initiated (87). Mercer et al. (83) showed for the first time that chimeric immunotolerant uPA-SCID mice were susceptible to HCV infection, thereby permitting the *in vivo* study of HCV biology and the evaluation of different antivirals. Efficient infection is independent from HCV genotype, but human albumin plasma levels exceeding 1 mg/ml are required for a consistent HCV infection in chimeric mice, whereas infectivity criteria for HBV infection are clearly less stringent (91, 92).

However, several shortcomings can be highlighted regarding the uPA-SCID mouse model: high neonatal lethality, a tendency to develop kidney disorders, lower body size, reduction of donor hepatocytes (even in homozygotes), less efficient breeding, technically challenging surgical manipulation in young and fragile mice, and finally the inability to expand engrafted hepatocytes (83, 88, 93–96). Tateno et al. (93) hypothesized that the first four mentioned limitations are caused by inadequate transgene structure and/or very high expression levels of the uPA gene before or after birth. Therefore, they produced chimeric mice using embryonic stem cell techniques in order to generate a number of transgenic lines. In addition, transgenic lines with the most appropriate uPA expression for a damaged, but not a detrimental liver were selected (93). This variant is called the hemizygous cDNA-uPA-SCID mouse model (93). More albumin-positive human hepatocytes are present compared to the original model, potentially due to an overgrowth of mouse hepatocytes in the uPA-SCID mouse by somatic deletion of uPA genes (97). After HBV infection, high titers of HBV viremia that persisted for at least 34 weeks are found in cDNA-uPA-SCID mice, but entecavir treatment results in a similar viremia decline in both models (97). HCV viremia is significantly more observed in cDNA-uPA-SCID mice in comparison with uPA-SCID mice, but not one mouse remains HCV-positive 8 weeks post-inoculation (97). Finally, fewer kidney disorders, higher body weight, and a higher survival rate are observed in the cDNA-uPA-SCID model (93, 97). Taken together, the cDNA-uPA-SCID mouse model may be preferred over the original uPA-SCID model for the study of HBV and HCV biology and by extent for the evaluation of anti-HBV/HCV drugs.

A second variant consists of transgenic mice carrying the uPA gene driven by the MUP promoter (98). These mice can be backcrossed onto a SCID/Beige background, resulting in the MUP-uPA SCID/Bg mouse model (99). The initial purpose of this model was to study liver regeneration after repopulation of the diseased liver, but Tesfaye et al. (100) were able to show that, upon humanization, these mice are susceptible to infection with HBV, genotypes 1–6 of HCV and tissue culture-derived virus (98, 99). Interestingly, these mice are in better health compared to the classical uPA-SCID mouse model and they offer a longer time window (up to 4–12 months of age) for transplantation of human hepatocytes (100). The same group (101) reported successful HCV infection after engraftment with hepatocyte-like cells, generated from both human embryonic stem cells and patient-specific human-induced pluripotent stem cells. Finally, this model is also valuable for the study of HCV-associated HCC and for the analysis of tumor-promoting factors in liver cancer (102).

As a third uPA-based variant, the non-obese diabetic (NOD)/Shi-scid IL2Rg^null^ (NOG) background is employed, resulting in the uPA/NOG mouse model (94). Donor hepatocytes can be transplanted in 6-week-old uPA/NOG mice which enable easier surgical manipulation and moreover an improved survival rate of the transplanted mice (94). In addition, absence of neonatal lethality increases the efficacy of homozygote production by mating and finally, the severely immunocompromised NOG background allows higher xenogeneic cell engraftment (94). Another advantage is that a relatively low frequency of physical loss of the transgene is observed (94). However, HCV infection is not reported in this model yet. Importantly, Hasegawa et al. (103) generated another model by using an alternative strategy for the endogenous liver injury: targeting the expression of herpes simplex virus type 1 thymidine kinase to the liver of the NOG mice. Hepatocytes that express this transgene can be ablated after brief exposure of a non-toxic dose of ganciclovir (103). Thereby, mouse livers can be stably replaced with mature and functional human liver tissue at a chosen time (103). This model can be successfully infected with HBV and HCV and is therefore useful to test different antiviral agents (104).

Taken together, the chimeric human liver uPA-SCID mouse model or discussed variants thereof have proven valuable for *in vivo* metabolism studies, basic biology research of HCV infection, and the evaluation of different antiviral therapies and passive immunization strategies (79, 105–113). Our group also contributed by demonstrating the prophylactic effect of monoclonal and polyclonal antibodies, isolated from a chronically infected patient, against challenge with different HCV genotypes (107, 108, 114). In addition, anti-receptor antibodies are shown to protect these mice from a subsequent challenge with HCV of different genotypes (106, 115–117). Next to chimpanzees, this human liver chimeric mouse model is also attractive for monitoring HCV drug resistance (118). Our laboratory has particular interest in this matter and showed that the combination of DAAs with entry inhibitors restricts the breakthrough of DAA-resistant viruses (119, 120). Finally, the uPA-SCID mouse model is also applicable for studies concerning malaria, which is caused by the parasite *Plasmodium falciparum*, and the study of the hepatitis E virus (HEV) (121–125).

### The FRG Mouse Model With Humanized Liver

In the original uPA-SCID mouse model, hepatocyte transplantation needs to be performed very shortly after birth (i.e., in very fragile and small animals) (126). Because of this practical inconvenience, other models were explored in which liver injury can be induced at a later age, such as in certain earlier discussed variants of the uPA-SCID model. Another example is based on mice that have a genetic knockout for fumarylacetoacetate hydrolase (Fah^−/−^), a metabolic enzyme that catalyzes the last step of the tyrosine catabolism pathway (127, 128). This knockout results in an accumulation of toxic compounds (e.g., fumarylacetoacetate and maleylacetoacetate), which in turn leads to liver dysfunction and lethality, unless mice are rescued by the protective drug 2-(2-nitro-4-trifluoromethylbenzoyl)-1,3-cyclohexanedione (NTBC) (127–129). NTBC blocks the enzyme hydroxyphenylpyruvate dioxygenase upstream of FAH, thus preventing the accumulation of hepatotoxic metabolites (130). Using this approach, Grompe et al. (129) showed that liver injury can be induced at any desired time point when NTBC is withdrawn. The resulting prolonged lifespan of these Fah^−/−^ mice resembles a phenotype of humans suffering with hereditary tyrosinaemia type I (HT1), which is an inborn error of metabolism caused by deficiency of the Fah enzyme (127–129). As a result, the adult Fah^−/−^ mouse, when removed from NTBC treatment, is a valuable model for studying the pathophysiology and evaluating the treatment options of HT1 and by extension hepatic cancer (128, 129). In the immunodeficient FRG mouse, the aforementioned Fah, RAG2, and common γ-chain of the interleukin receptor (Il2rg) are knocked out. The livers of these mice can be successfully repopulated with human hepatocytes after NTBC withdrawal (95, 130). In support of enhanced engraftment, Azuma et al. (95) administered an uPA-expressing adenovirus before transplantation which induces cell-autonomous hepatotoxicity rendering a more favorable niche for transplantation (131). In later experiments, Bissig et al. (96) showed higher transplantation rates (up to 95%) using an increased human hepatocyte dose per mouse. They also demonstrated successful infection of the FRG mouse with HBV and HCV, however, infection with HCV is only achieved in mice with a high human liver chimerism (96).

The FRG mouse model is in some ways favorable over the uPA-SCID mouse model. First, the deficiency caused by the Fah deletion cannot revert back to its wild type form, as seen in the uPA-SCID model (95). As a result, serial transplantations are possible and transplantation can be performed in adult animals (at any age) which simplifies surgery (95). Second, mutant breeders are completely viable and finally, there is no renal disease observed (95). Unfortunately, there are also drawbacks for such a model. First, primary engraftment does not occur in 100% of the recipients, even when the aforementioned urokinase-expressing adenovirus is administered (95). Second, the growth disadvantage of mouse hepatocytes in the FRG mouse depends on the absorbed tyrosine and the use of NTBC, whereas the growth advantage in uPA-SCID mice is sustained as long as the transgene is expressed (130).

Applications of this FRG mouse model with humanized liver are wide-ranging. First, human lipid and bile acid metabolism can be studied, next to the metabolism of candidate pharmaceuticals or toxicity of drug metabolites (95, 132). Second, after inoculation with pathogens that are dependent on human liver cells for replication such as HBV, HCV, and HEV, the life cycle can be studied, but also experimental treatment options can be evaluated (95, 133–137). Finally, because the FRG mouse model also supports complete *P. falciparum* liver stage development, this model is suitable for evaluating existing drugs and screening of candidate antimalarials (138).

## Immunocompetent Xenograft Mouse Models

The human liver xenograft mouse models are very valuable as challenge models for HCV or other human hepatotropic pathogens, but their major drawback is the lack of a functional immune system. As a consequence, they cannot be used for the study of HCV-specific immune responses or HCV immunopathogenesis after infection, nor for HCV vaccine studies (79). Second, histopathology such as fibrosis, cirrhosis, or HCC has not been reported, in contrast to what is seen in humans that are chronically infected with HCV (75). In human patients, an ongoing inflammatory response is probably responsible for disease progression, so the presence of a functional human immune system in HCV mouse models is highly demanded and explored (75).

### The Tolerized Rat Model

Another way to avoid rejection of allogeneic transplants, in addition to generalized immunosuppression or the use of genetically immunodeficient animals, is by induction of immunological tolerance to transplanted cells in immunocompetent animals (76, 77, 85, 139, 140). Therefore, Huh7 cells can be injected *in utero* into the peritoneal cavity of fetal rats (139). In this time frame, the immune system is still in development, so tolerance toward engrafted hepatocytes can be established (139, 140). Corresponding cells are then intrasplenically injected into the newborn rats within the first 24 h after birth (139). The major benefit of this model is that there is no need for genetic or pharmacological immunosuppression (139). However, engraftment rates are much lower compared to the uPA-SCID model for example, because there is no mechanism for host hepatocyte depletion (141). The use of hepatoma cells instead of primary hepatocytes also limits further applications. Another drawback is the mismatch between the human major histocompatibility complex (MHC) antigens on the transplanted cells and the rat immune system, so there will be no recognition of HCV antigens by the immune cells of the rat (141). Despite these limitations, HCV gene expression, viral replication, and hepatitis symptoms can be observed when these tolerized immunocompetent rats are intrasplenically injected with HCV inocula 1 week after transplantation (141). Unfortunately, HCV viremia is low (peak at 22,500 copies/ml) and the observed inflammation is probably due to cytokine-mediated effects (141).

### The Dually (Immune System and Hepatocytes) Engrafted Mouse Models

To overcome the human/rodent MHC mismatch as in the tolerized rat model, it would be favorable to introduce both human hepatocytes and human immune cells from the same donor into the same recipient animal. The first mouse model that supported this double engraftment was generated in 2011 (142). A fusion protein of the FK506 binding protein (FKBP) and caspase 8 under the control of the albumin promoter (AFC8) is therefore transgenically expressed in hepatocytes of immunodeficient Balb/C Rag2-γC^null^ mice. After administration of an FKBP dimerizer, hepatocytes that expressed the transgene are depleted (142, 143). This induced liver-specific cell death provides a niche for engraftment with human hepatocyte progenitors (142). Moreover, after irradiation, these mice are transplanted with human CD34^+^ hematopoietic stem cells (HSCs) from the same human fetal liver tissue, providing these AFC8-hu HSC/Hep mice with a, to some extent, functional human immune system (142, 144). Following inoculation with primary HCV isolates, HCV infection can be observed that in turn induces infiltration of human immune cells in the livers with liver inflammation and fibrosis as a result (142, 145). This model enables the study of HCV-specific immune responses (i.e., T-cell responses) and HCV immunopathogenesis (142, 145). However, HCV RNA could only be detected in the liver and not in plasma, probably due to the low level of human liver engraftment (~15%) in this model (126, 142). Another limitation is the suboptimal activity of the immune system inherent in human CD34^+^ HSC transplanted mice and also the lack of functional B-lymphocytes that hampers the study of antibody responses and vaccine development (126, 142, 146).

A second immunocompetent model was launched by Gutti et al. (147) who used non-myeloablative conditioning with treosulfan as a safe and well-tolerated alternative to total body irradiation for HSC transplantation. Long-term dual reconstitution is achieved in uPA/NOG mice with HSCs and allogeneic mature hepatocytes (not fetal hepatoblasts) (147). Even MHC mismatched transplantation is sustained without any evidence of hepatocyte rejection by the human immune system (147). Wilson et al. (148) also accomplished double humanization of mice. Following preconditioning with a DNA-damaging chemical for enhanced HSC engraftment and an uPA-expressing adenovirus for enhanced hepatocyte engraftment, they co-transplanted adult human hepatocytes and HSCs in immunodeficient FRG mice on a NOD-strain background (FRGN mice) (95, 148). Another variant is achieved in BALB/c RAG2^−/−^ IL-2Rγc^−/−^ NOD.sirpα (BRGS) mice that harbor the uPA transgene (uPA/BRGS mice) (149). Irradiated newborn pups are injected with human HSCs and later implanted with human hepatocytes to generate dually engrafted mice that are not haplo-type matched. Engraftment (~20–50% of chimerism) is stable for at least 5 months and is similar as observed in the uPA/NOG and FRGN host, but higher than in the AFC8 host (142, 147–149). Interestingly, a complete viral life cycle can be observed after HBV infection in this model (150). This enables the evaluation of experimental anti-HBV therapies, but also the study of anti-HBV immune responses (150). Bility et al. (151) also reported successful HBV infection in a similar human liver progenitor cell and human CD34^+^ HSC cell engraftment model using mice on a NOD-SCID IL2rγ^−/−^ background (HLA-A2/NSG mice). These mice carry the human HLA-A2 transgene that enhances the development of human MHC-restricted T-lymphocytes (151, 152). To promote efficient hepatocyte repopulation, mice are first treated with an anti-Fas agonistic antibody (151, 153). Chen et al. (154) performed one-step engraftment of hepatoblasts and a matching human immune system using fetal liver-derived HSC cells in the same NSG mouse (human immune system and liver or HIL mice) and this without the need for transgenic modification or drug treatment. HIL mice support HCV infection, liver inflammation, human HCV-specific immune responses, as well as liver fibrosis, however, in a low number of hepatocytes (154, 155). This can be explained by the low human chimerism rate (<10%) (154, 155). Antiviral treatment using IFNα-2a is able to block the progression of the HCV-associated liver pathogenesis (154, 155). These HCV-infected mice also show expansion of monocytes/macrophages and (especially CD4^+^) T-cells, suggesting exhaustion of immune cell phenotypes as seen in HCV patients (156). Unfortunately, HCV infection is not reported in every discussed dually engrafted model and this will also be challenging, especially due to the very low engraftment rates.

## Viral Adaptation

Hepatitis C virus exhibits a narrow species tropism which is incompletely understood. Resistance of mice to HCV infection is determined to be at the level of viral entry and/or replication. There are two ways of surmounting this barrier: either the host can be adapted to the virus or the virus can be adapted to the host. First, utilizing the error-prone replication of RNA viruses, the HCV virus can be adapted to the murine environment. More specifically, long-term cultivation in the presence of mouse cells could allow the virus to adapt to murine entry factors (CD81, OCLN, CLDN1, and SR-BI). Bitzegeio et al. (157) attempted to adapt an HCV genotype 2a strain (Jc1) to the murine CD81. They identified three adaptive mutations in the HCV envelope proteins E1 and E2. This Jc1/mCD81 virus has increased affinity for the extracellular loops of human CD81, indicating a more accessible binding site for human CD81 (157). The uptake of this murine-tropic HCV in mouse primary hepatocytes *in vitro* and *in vivo* is rather inefficient and more modifications are required to increase efficiency. There is unfortunately no persistent infection observed, even in mice with impaired innate and adaptive immune system. To conclude, additional barriers may exist in the replication and post-entry steps (158). In addition, the applicability of such systems for the study of entry processes might be affected by the influence of the adaptive mutations on the envelope conformation and receptor usage.

## Genetically Humanized Mouse Model

Rather than to adapt the virus to a new host, an alternative strategy could be to genetically adapt the host to natural HCV isolates. Despite differences to the natural human host of HCV, an immunocompetent animal model can be generated in this way. Transient expression of the minimal human factors (CD81, OCLN, CLDN1, and SR-BI) by adenoviral delivery in Rosa26-Fluc mice allows entry of HCVcc in mouse hepatocytes (35). Furthermore, mice transgenic for these four human receptors (4hEF-mice), but with deficiencies in several innate immune signaling pathways (STAT1^−/−^), support not only viral entry of HCVcc but also low-level replication and sustained HCV infection for 90 days. The infection elicits antiviral cellular and humoral responses, but does not result in development of liver disease (159).

However, these models express non-physiologically high levels of the entry factors and impair tight junction formation and B-cell development (160). Interestingly, by selectively humanizing the second extracellular loops of CD81 and OCLN, required for HCVcc entry, the chimeric alleles are expressed at physiological levels and mice support HCV uptake at similar levels as mice expressing HCV receptors using transgenical or adenoviral methods. Also, tight junctions are formed normally and the defects in B-cell development are absent (160).

However, since replication in immunocompetent mice is inefficient, the latter model does not allow a profound study of all complex virus–host interactions. Viral RNA replication in mouse cells appears to be the final hurdle to overcome in order to reconstitute the entire viral life cycle in mice. Chen et al. (161) described an immunocompetent animal model permissive for HCV infection and ensuing development of liver disease. They created transgenic mice expressing OCLN and CD81 on an outbred ICR (CD-1) background (C/OTg). These mice can be infected with serum- or cell culture-derived HCV and sustain this infection for over 12 months. Moderate hepatic inflammation, micro- and macro-vesicular steatosis, and fibrosis are observed in some of the infected animals. However, none of the animals develop HCC (161). It is rather striking that HCV can establish a persistent infection in ICR-C/OTg mice, whereas a similar approach on a C57BL/6 background fails to show sustained HCV replication. Backcrossing C/OTg to a C57BL/6 background (B6-C/OTg) significantly reduces the RNA copy number in serum and liver. ICR hepatocytes express higher levels of apolipoprotein E, which is shown to improve HCV production (162). Also, miR-122 is not upregulated upon HCV infection in B6-C/OTg (161). In conclusion, the ICR-C/OTg mouse model appears to fulfill to a large extent the criteria for a suitable HCV animal model and is therefore a valuable addition to the current pool of animal models.

## HCV Homologs

Alternative models are based on the use of HCV homologs. These HCV-related viruses infect either rodents, horses or dogs and can therefore be used to study viral biology, pathogenesis, and host immune responses in an immunocompetent setting. The GB virus B has long been the only known homolog to HCV. This virus was first discovered in tamarins experimentally infected with serum of a surgeon (G.B.) suffering from acute hepatitis (163). The infected tamarins developed acute hepatitis (164) and are used together with marmosets as a surrogate model for the study of protective immunity (165) and evaluation of antivirals (166). However, persistence is rare in these animals and the natural host is yet to be identified (163, 167, 168).

By using deep sequencing virome analyses, novel HCV-related hepaciviruses and pegiviruses have been identified in dogs, horses, bats, rodents, and non-human primates (168). Several of these viruses have the potential to serve as a surrogate model for HCV. However, not all are hepatotropic or mimic the natural course of HCV infection. The non-primate hepacivirus (NPHV) was first discovered in dogs and therefore termed canine hepacivirus (169), but subsequent studies revealed that horses are the natural host for this virus (168). NPHV infection in horses greatly resembles HCV infection in humans. It is a hepatotropic virus that is able to establish a persistent infection, although the chronicity rate is considerably lower than for HCV infection. The host immune response is similar to that in humans, including the delayed seroconversion and immune-related liver pathology (168). These characteristics allow NPHV to be a valuable animal model for HCV, especially since it is immunocompetent. Drawbacks, however, are the large size and animal care costs that accompany this model. Conceivably, rodents are still the desired animal model, due to their small size, easy handling, and possibility to be genetically manipulated. Therefore, the newly discovered rodent hepaciviruses (RHV) are of particular interest (170–172). Infections of these viruses in their natural host, or possibly in immunocompetent laboratory inbred mouse strains, require further investigation.

Methodical searches for hepaciviruses in several wild rodent species have led to the identification of potential small animal models for HCV. Some of these rodents, including bank voles (*Myodes glareolus*) and rats (*Rattus norvegicus*), experience signs of liver inflammation after infection with a RHV. During a metagenomics survey in commensal Norway rats (Nr) in New York city, Firth et al. (172) also discovered some new viruses, including two novel hepaciviruses (NrHV-1 and NrHV-2) and one novel pegivirus (NrPgV). These hepaciviruses were demonstrated to be hepatotropic and are consequently the first small-mammal hepaciviruses known to replicate in the liver (172). Although rats are the natural hosts of NrHV, Billerbeck et al. (173) aimed to develop a mouse model of NrHV infection. NrHV is able to establish a persistent infection in immunocompromised mice lacking type I interferon and adaptive immunity (A129, AG129, and NRG). On the other hand, immunocompetent mice (C57BL/6J and BALB/c) clear the virus in a few weeks (173). NrHV, passaged through NRG mice, is cleared significantly slower than NrHV derived from rats, indicating possible adaptation to the mouse host. The developed immunocompetent inbred mouse model can potentially help to unravel certain mechanisms of hepacivirus host adaptation, immune activation and evasion, and development of liver disease (173). Because this inbred mouse model only results in an acute, self-limiting infection, Trivedi et al. (174) searched for a fully immunocompetent surrogate model in which a persistent infection can be established. The natural host of NrHV, the rat, was therefore further investigated. Inbred Brown Norway rats fail to even partially control the infection, while different outbred lines [Spraque–Dawley, Holtzman (HTZ), Long Evan, and Wistar Han] show limited suppression of viral replication. HTZ rats display the largest suppression of viremia and were explored in more detail. The rats exhibit hepatic inflammation characterized by dense lymphocytic aggregates focused on the portal tracts, parenchymal damage, associated with apoptotic hepatocytes and macro- and micro-vesicular steatosis, characteristic for human HCV infection (174). This model is also suitable to study the role of various interferon stimulating genes and immune responses in hepacivirus pathogenesis. Thus, NrHV infected rats can serve as an informative, fully immunocompetent surrogate to study the mechanisms of HCV persistence, immunity, and pathogenesis.

## Conclusion

Despite extensive research, there is still no vaccine available for the prevention of HCV infection. In order to design and test new vaccines, the immunocompetent human liver xenograft mouse models are very promising. Next to the study of HCV immune responses, these models also allow investigation of disease progression. Contrary to this, the immunocompromised human liver xenograft mouse models only allow passive immunization. In this way, antibodies targeting different genotypes of HCV can be evaluated. Vaccine studies are not relevant, because these mice lack or only show limited cellular immunity. Furthermore, during the past decade, especially the uPA-based mouse with humanized liver has considerably contributed to our understanding of the HCV life cycle and the development of antiviral strategies. Alternatively, for studying the basic aspects of HCV biology, viral replication or for the evaluation of certain antiviral strategies, it may not be necessary to establish complicated dual-chimeric models. The genetically humanized models are adequate to study viral biology. However, they can only be used to evaluate prophylactic vaccines, not therapeutic vaccines. Finally, the HCV homologs, more specifically NrHV, can be used for vaccination studies and for the evaluation of both humoral and cellular immune responses. The knowledge that is built from this model can be partially transferred to the existing HCV models, but it is important to consider that HCV and hepaciviruses comprise different viruses. In conclusion, it is clear that the HCV model of choice is highly dependent upon the specific research question. The development and characterization of new HCV animal models or the improvement of existing models, especially those with a human immune system, is highly demanded to develop a potent HCV vaccine. An effective vaccine is probably the most essential key for eradication of HCV.

## Author Contributions

RB and LC wrote the manuscript. AM and PM revised the manuscript.

## Conflict of Interest Statement

The authors declare that the research was conducted in the absence of any commercial or financial relationships that could be construed as a potential conflict of interest. The handling Editor declared a past co-authorship with one of the authors (PM).

## References

[1] Lanford RE Walker CM Lemon SM. The Chimpanzee model of viral hepatitis: advances in understanding the immune response and treatment of viral hepatitis. ILAR J (2017) 58(2):172–89. 10.1093/ilar/ilx028 29045731 PMC5886334

[2] Beinhardt S Al-Zoairy R Kozbial K Stattermayer AF Maieron A Stauber R Long-term follow-up of ribavirin-free DAA-based treatment in HCV recurrence after orthotopic liver transplantation. Liver Int (2017). 10.1111/liv.13652 29197145

[3] Kumthip K Maneekarn N. The role of HCV proteins on treatment outcomes. Virol J (2015) 12:217. 10.1186/s12985-015-0450-x 26666318 PMC4678629

[4] Lohmann V Körner F Koch JO Herian U Theilmann L Bartenschlager R. Replication of subgenomic hepatitis C virus RNAs in a hepatoma cell line. Science (1999) 285(5424):110–3. 10.1126/science.285.5424.110 10390360

[5] Blight KJ Kolykhalov AA Rice CM. Efficient initiation of HCV RNA replication in cell culture. Science (2000) 290(5498):1972–4. 10.1126/science.290.5498.1972 11110665

[6] Foy E Li K Wang C Sumpter R Ikeda M Lemon SM Regulation of interferon regulatory factor-3 by the hepatitis C virus serine protease. Science (2003) 300(5622):1145–8. 10.1126/science.1082604 12702807

[7] Meylan E Curran J Hofmann K Moradpour D Binder M Bartenschlager R Cardif is an adaptor protein in the RIG-I antiviral pathway and is targeted by hepatitis C virus. Nature (2005) 437(7062):1167. 10.1038/nature04193 16177806

[8] Bartenschlager R Lohmann V Penin F. The molecular and structural basis of advanced antiviral therapy for hepatitis C virus infection. Nat Rev Microbiol (2013) 11(7):482–96. 10.1038/nrmicro3046 23748342

[9] Baumert TF Vergalla J Satoi J Thomson M Lechmann M Herion D Hepatitis C virus-like particles synthesized in insect cells as a potential vaccine candidate. Gastroenterology (1999) 117(6):1397–407. 10.1016/S0016-5085(99)70290-8 10579981

[10] Buonocore L Blight KJ Rice CM Rose JK. Characterization of vesicular stomatitis virus recombinants that express and incorporate high levels of hepatitis C virus glycoproteins. J Virol (2002) 76(14):6865–72. 10.1128/JVI.76.14.6865-6872.2002 12072487 PMC136334

[11] Flint M Thomas JM Maidens CM Shotton C Levy S Barclay WS Functional analysis of cell surface-expressed hepatitis C virus E2 glycoprotein. J Virol (1999) 73(8):6782–90. 10400776 10.1128/jvi.73.8.6782-6790.1999 PMC112763

[12] Lagging LM Meyer K Owens RJ Ray R. Functional role of hepatitis C virus chimeric glycoproteins in the infectivity of pseudotyped virus. J Virol (1998) 72(5):3539–46. 9557633 10.1128/jvi.72.5.3539-3546.1998 PMC109573

[13] Matsuura Y Tani H Suzuki K Kimura-Someya T Suzuki R Aizaki H Characterization of pseudotype VSV possessing HCV envelope proteins. Virology (2001) 286(2):263–75. 10.1006/viro.2001.0971 11485395

[14] Hsu M Zhang J Flint M Logvinoff C Cheng-Mayer C Rice CM Hepatitis C virus glycoproteins mediate pH-dependent cell entry of pseudotyped retroviral particles. Proc Natl Acad Sci U S A (2003) 100(12):7271–6. 10.1073/pnas.0832180100 12761383 PMC165865

[15] Bartosch B Dubuisson J Cosset F-L Infectious hepatitis C virus pseudo-particles containing functional E1–E2 envelope protein complexes. J Exp Med (2003) 197(5):633–42. 10.1084/jem.20021756 12615904 PMC2193821

[16] Drummer HE Maerz A Poumbourios P. Cell surface expression of functional hepatitis C virus E1 and E2 glycoproteins. FEBS Lett (2003) 546(2–3):385–90. 10.1016/S0014-5793(03)00635-5 12832074

[17] Kato T Furusaka A Miyamoto M Date T Yasui K Hiramoto J Sequence analysis of hepatitis C virus isolated from a fulminant hepatitis patient*. J Med Virol (2001) 64(3):334–9. 10.1002/jmv.1055 11424123

[18] Lindenbach BD Evans MJ Syder AJ Wölk B Tellinghuisen TL Liu CC Complete replication of hepatitis C virus in cell culture. Science (2005) 309(5734):623–6. 10.1126/science.1114016 15947137

[19] Wakita T Pietschmann T Kato T Date T Miyamoto M Zhao Z Production of infectious hepatitis C virus in tissue culture from a cloned viral genome. Nat Med (2005) 11(7):791–6. 10.1038/nm0805-905b 15951748 PMC2918402

[20] Zhong J Gastaminza P Cheng G Kapadia S Kato T Burton DR Robust hepatitis C virus infection in vitro. Proc Natl Acad Sci U S A (2005) 102(26):9294–9. 10.1073/pnas.0503596102 15939869 PMC1166622

[21] von Schaewen M Ploss A. Murine models of hepatitis C: what can we look forward to? Antiviral Res (2014) 104:15–22. 10.1016/j.antiviral.2014.01.007 24462693 PMC4068254

[22] Frentzen A Anggakusuma Gurlevik E Hueging K Knocke S Ginkel C Cell entry, efficient RNA replication, and production of infectious hepatitis C virus progeny in mouse liver-derived cells. Hepatology (2014) 59(1):78–88. 10.1002/hep.26626 23873628

[23] Barth H Schafer C Adah MI Zhang F Linhardt RJ Toyoda H Cellular binding of hepatitis C virus envelope glycoprotein E2 requires cell surface heparan sulfate. J Biol Chem (2003) 278(42):41003–12. 10.1074/jbc.M302267200 12867431

[24] Agnello V Abel G Elfahal M Knight GB Zhang QX. Hepatitis C virus and other flaviviridae viruses enter cells via low density lipoprotein receptor. Proc Natl Acad Sci U S A (1999) 96(22):12766–71. 10.1073/pnas.96.22.12766 10535997 PMC23090

[25] Pileri P Uematsu Y Campagnoli S Galli G Falugi F Petracca R Binding of hepatitis C virus to CD81. Science (1998) 282(5390):938–41. 10.1126/science.282.5390.938 9794763

[26] Scarselli E Ansuini H Cerino R Roccasecca RM Acali S Filocamo G The human scavenger receptor class B type I is a novel candidate receptor for the hepatitis C virus. EMBO J (2002) 21(19):5017–25. 10.1093/emboj/cdf529 12356718 PMC129051

[27] Evans MJ von Hahn T Tscherne DM Syder AJ Panis M Wölk B Claudin-1 is a hepatitis C virus co-receptor required for a late step in entry. Nature (2007) 446(7137):801–5. 10.1038/nature05654 17325668

[28] Liu S Yang W Shen L Turner JR Coyne CB Wang T. Tight junction proteins claudin-1 and occludin control hepatitis C virus entry and are downregulated during infection to prevent superinfection. J Virol (2009) 83(4):2011–4. 10.1128/JVI.01888-08 19052094 PMC2643775

[29] Ploss A Evans MJ Gaysinskaya VA Panis M You H de Jong YP Human occludin is a hepatitis C virus entry factor required for infection of mouse cells. Nature (2009) 457(7231):882–6. 10.1038/nature07684 19182773 PMC2762424

[30] Lupberger J Zeisel MB Xiao F Thumann C Fofana I Zona L EGFR and EphA2 are host factors for hepatitis C virus entry and possible targets for antiviral therapy. Nat Med (2011) 17(5):589–95. 10.1038/nm.2341 21516087 PMC3938446

[31] Sainz B Barretto N Martin DN Hiraga N Imamura M Hussain S Identification of the Niemann-Pick C1-like 1 cholesterol absorption receptor as a new hepatitis C virus entry factor. Nat Med (2012) 18(2):281–5. 10.1038/nm.2581 22231557 PMC3530957

[32] Martin DN Uprichard SL. Identification of transferrin receptor 1 as a hepatitis C virus entry factor. Proc Natl Acad Sci U S A (2013) 110(26):10777–82. 10.1073/pnas.1301764110 23754414 PMC3696786

[33] Wu X Lee EM Hammack C Robotham JM Basu M Lang J Cell death-inducing DFFA-like effector b is required for hepatitis C virus entry into hepatocytes. J Virol (2014) 88(15):8433–44. 10.1128/JVI.00081-14 24829338 PMC4135929

[34] Li Q Sodroski C Lowey B Schweitzer CJ Cha H Zhang F Hepatitis C virus depends on E-cadherin as an entry factor and regulates its expression in epithelial-to-mesenchymal transition. Proc Natl Acad Sci U S A (2016) 113(27):7620–5. 10.1073/pnas.1602701113 27298373 PMC4941495

[35] Dorner M Horwitz JA Robbins JB Barry WT Feng Q Mu K A genetically humanized mouse model for hepatitis C virus infection. Nature (2011) 474(7350):208. 10.1038/nature10168 21654804 PMC3159410

[36] Choo QL Kuo G Weiner AJ Overby LR Bradley DW Houghton M. Isolation of a cDNA clone derived from a blood-borne non-A, non-B viral hepatitis genome. Science (1989) 244(4902):359–62. 10.1126/science.2523562 2523562

[37] Lanford RE Bigger C Bassett S Klimpel G. The chimpanzee model of hepatitis C virus infections. ILAR J (2001) 42(2):117–26. 10.1093/ilar.42.2.117 11406714

[38] Muchmore E Popper H Peterson DA Miller MF Lieberman HM. Non-A, non-B hepatitis-related hepatocellular carcinoma in a chimpanzee. J Med Primatol (1988) 17(5):235–46. 3148034

[39] Shimizu YK Weiner AJ Rosenblatt J Wong DC Shapiro M Popkin T Early events in hepatitis C virus infection of chimpanzees. Proc Natl Acad Sci U S A (1990) 87(16):6441–4. 10.1073/pnas.87.16.6441 2117282 PMC54550

[40] Choi Y Dienes H-P Krawczynski K. Kinetics of miR-122 expression in the liver during acute HCV infection. PLoS One (2013) 8(10):e76501. 10.1371/journal.pone.0076501 24124569 PMC3790687

[41] Choi YH Jin N Kelly F Sakthivel SK Yu T Elevation of alanine aminotransferase activity occurs after activation of the cell-death signaling initiated by pattern-recognition receptors but before activation of cytolytic effectors in NK or CD8+T cells in the liver during acute HCV infection. PLoS One (2016) 11(10):e0165533 10.1371/journal.pone.0165533 27788241 PMC5082795

[42] Cooper S Erickson AL Adams EJ Kansopon J Weiner AJ Chien DY Analysis of a successful immune response against hepatitis C virus. Immunity (1999) 10(4):439–49. 10.1016/S1074-7613(00)80044-8 10229187

[43] Shoukry NH Sidney J Sette A Walker CM. Conserved hierarchy of helper T cell responses in a chimpanzee during primary and secondary hepatitis C virus infections. J Immunol (2004) 172(1):483–92. 10.4049/jimmunol.172.1.483 14688358

[44] Folgori A Capone S Ruggeri L Meola A Sporeno E Ercole BB A T-cell HCV vaccine eliciting effective immunity against heterologous virus challenge in chimpanzees. Nat Med (2006) 12(2):190–7. 10.1038/nm1353 16462801

[45] Bukh J Forns X Emerson SU Purcell RH. Studies of hepatitis C virus in chimpanzees and their importance for vaccine development. Intervirology (2001) 44(2–3):132–42. 10.1159/000050040 11509874

[46] Morin TJ Broering TJ Leav BA Blair BM Rowley KJ Boucher EN Human monoclonal antibody HCV1 effectively prevents and treats HCV infection in chimpanzees. PLoS Pathog (2012) 8(8):e1002895. 10.1371/journal.ppat.1002895 22952447 PMC3431327

[47] Coburn CA Meinke PT Chang W Fandozzi CM Graham DJ Hu B Discovery of MK-8742: an HCV NS5A inhibitor with broad genotype activity. ChemMedChem (2013) 8(12):1930–40. 10.1002/cmdc.201300343 24127258

[48] Carroll SS Ludmerer S Handt L Koeplinger K Zhang NR Graham D Robust antiviral efficacy upon administration of a nucleoside analog to hepatitis C virus-infected chimpanzees. Antimicrob Agents Chemother (2009) 53(3):926–34. 10.1128/AAC.01032-08 19075052 PMC2650549

[49] Chen C-M He Y Lu L Lim HB Tripathi RL Middleton T Activity of a potent hepatitis C virus polymerase inhibitor in the chimpanzee model. Antimicrob Agents Chemother (2007) 51(12):4290–6. 10.1128/AAC.00723-07 17908950 PMC2167986

[50] Olsen DB Davies M-E Handt L Koeplinger K Zhang NR Ludmerer SW Sustained viral response in a hepatitis C virus-infected chimpanzee via a combination of direct-acting antiviral agents. Antimicrob Agents Chemother (2011) 55(2):937–9. 10.1128/AAC.00990-10 21115793 PMC3028818

[51] Sourisseau M Goldman O He W Gori JL Kiem HP Gouon-Evans V Hepatic cells derived from induced pluripotent stem cells of pigtail macaques support hepatitis C virus infection. Gastroenterology (2013) 145(5):966–9.e7. 10.1053/j.gastro.2013.07.026 23891978 PMC3805793

[52] Scull MA Shi C de Jong YP Gerold G Ries M von Schaewen M Hepatitis C virus infects rhesus macaque hepatocytes and simianized mice. Hepatology (2015) 62(1):57–67. 10.1002/hep.27773 25820364 PMC4482775

[53] Abe K Kurata T Teramoto Y Shiga J Shikata T. Lack of susceptibility of various primates and woodchucks to hepatitis C virus. J Med Primatol (1993) 22(7–8):433–4. 8169947

[54] Amako Y Tsukiyama-Kohara K Katsume A Hirata Y Sekiguchi S Tobita Y Pathogenesis of hepatitis C virus infection in *Tupaia belangeri*. J Virol (2010) 84(1):303–11. 10.1128/JVI.01448-09 19846521 PMC2798454

[55] Feng Y Feng Y-M Lu C Han Y Liu L Sun X Tree shrew, a potential animal model for hepatitis C, supports the infection and replication of HCV in vitro and in vivo. J Gen Virol (2017) 98(8):2069–78. 10.1099/jgv.0.000869 28758632 PMC5656785

[56] Ding CB Zhao Y Zhang JP Peng ZG Song DQ Jiang JD. A zebrafish model for subgenomic hepatitis C virus replication. Int J Mol Med (2015) 35(3):791–7. 10.3892/ijmm.2015.2063 25572289

[57] Rekha RD Amali AA Her GM Yeh YH Gong HY Hu SY Thioacetamide accelerates steatohepatitis, cirrhosis and HCC by expressing HCV core protein in transgenic zebrafish *Danio rerio*. Toxicology (2008) 243(1–2):11–22. 10.1016/j.tox.2007.09.007 17997003

[58] Frelin L Brenndörfer ED Ahlén G Weiland M Hultgren C Alheim M The hepatitis C virus and immune evasion: non-structural 3/4A transgenic mice are resistant to lethal tumour necrosis factor α mediated liver disease. Gut (2006) 55(10):1475–83. 10.1136/gut.2005.085050 16527836 PMC1856439

[59] Koike K Moriya K Ishibashi K Matsuura Y Suzuki T Saito I Expression of hepatitis C virus envelope proteins in transgenic mice. J Gen Virol (1995) 76(12):3031–8. 10.1099/0022-1317-76-12-3031 8847508

[60] Kawamura T Furusaka A Koziel MJ Chung RT Wang TC Schmidt EV Transgenic expression of hepatitis C virus structural proteins in the mouse. Hepatology (1997) 25(4):1014–21. 10.1002/hep.510250437 9096613

[61] Pasquinelli C Shoenberger JM Chung J Chang KM Guidotti LG Selby M Hepatitis C virus core and E2 protein expression in transgenic mice. Hepatology (1997) 25(3):719–27. 10.1002/hep.510250338 9049225

[62] Moriya K Yotsuyanagi H Shintani Y Fujie H Ishibashi K Matsuura Y Hepatitis C virus core protein induces hepatic steatosis in transgenic mice. J Gen Virol (1997) 78(7):1527–31. 10.1099/0022-1317-78-7-1527 9225025

[63] Moriya K Fujie H Shintani Y Yotsuyanagi H Tsutsumi T Ishibashi K The core protein of hepatitis C virus induces hepatocellular carcinoma in transgenic mice. Nat Med (1998) 4(9):1065. 10.1038/2053 9734402

[64] Kamegaya Y Hiasa Y Zukerberg L Fowler N Blackard JT Lin W Hepatitis C virus acts as a tumor accelerator by blocking apoptosis in a mouse model of hepatocarcinogenesis. Hepatology (2005) 41(3):660–7. 10.1002/hep.20621 15723444

[65] Tanaka N Moriya K Kiyosawa K Koike K Aoyama T. Hepatitis C virus core protein induces spontaneous and persistent activation of peroxisome proliferator-activated receptor alpha in transgenic mice: implications for HCV-associated hepatocarcinogenesis. Int J Cancer (2008) 122(1):124–31. 10.1002/ijc.23056 17764115

[66] Klopstock N Katzenellenbogen M Pappo O Sklair-Levy M Olam D Mizrahi L HCV tumor promoting effect is dependent on host genetic background. PLoS One (2009) 4(4):e5025. 10.1371/journal.pone.0005025 19340302 PMC2660413

[67] Sekiguchi S Kimura K Chiyo T Ohtsuki T Tobita Y Tokunaga Y Immunization with a recombinant vaccinia virus that encodes nonstructural proteins of the hepatitis C virus suppresses viral protein levels in mouse liver. PLoS One (2012) 7(12):e51656. 10.1371/journal.pone.0051656 23284733 PMC3524174

[68] Ahlén G Nyström J Pult I Frelin L Hultgren C Sällberg M In vivo clearance of hepatitis C virus nonstructural 3/4A–expressing hepatocytes by DNA vaccine-primed cytotoxic T lymphocytes. J Infect Dis (2005) 192(12):2112–6. 10.1086/498218 16288375

[69] Chen A Ahlén G Brenndörfer ED Brass A Holmström F Chen M Heterologous T cells can help restore function in dysfunctional hepatitis C virus nonstructural 3/4A-specific T cells during therapeutic vaccination. J Immunol (2011) 186(9):5107–18. 10.4049/jimmunol.1001790 21430225

[70] Lerat H Honda M Beard MR Loesch K Sun J Yang Y Steatosis and liver cancer in transgenic mice expressing the structural and nonstructural proteins of hepatitis C virus. Gastroenterology (2002) 122(2):352–65. 10.1053/gast.2002.31001 11832450

[71] Chouteau P Defer N Florimond A Caldéraro J Higgs M Gaudin A Hepatitis C virus (HCV) protein expression enhances hepatic fibrosis in HCV transgenic mice exposed to a fibrogenic agent. J Hepatol (2012) 57(3):499–507. 10.1016/j.jhep.2012.04.019 22613003

[72] Bumgardner GL Heininger M Li J Xia D Parker-Thornburg J Ferguson RM A functional model of hepatocyte transplantation for in vivo immunologic studies. Transplantation (1998) 65(1):53–61. 10.1097/00007890-199801150-00011 9448144

[73] Bumgardner GL Li J Heininger M Ferguson RM Orosz CG. In vivo immunogenicity of purified allogeneic hepatocytes in a murine hepatocyte transplant model. Transplantation (1998) 65(1):47–52. 10.1097/00007890-199801150-00010 9448143

[74] Bumgardner GL Li J Prologo JD Heininger M Orosz CG. Patterns of immune responses evoked by allogeneic hepatocytes: evidence for independent co-dominant roles for CD4+ and CD8+ T-cell responses in acute rejection. Transplantation (1999) 68(4):555–62. 10.1097/00007890-199908270-00019 10480416

[75] von Schaewen M Ding Q Ploss A. Visualizing hepatitis C virus infection in humanized mice. J Immunol Methods (2014) 410:50–9. 10.1016/j.jim.2014.03.006 24642425 PMC4163068

[76] Galun E Burakova T Ketzinel M Lubin I Shezen E Kahana Y Hepatitis C virus viremia in SCID – >BNX mouse chimera. J Infect Dis (1995) 172(1):25–30. 10.1093/infdis/172.1.25 7797923

[77] Ilan E Burakova T Dagan S Nussbaum O Lubin I Eren R The hepatitis B virus-trimera mouse: a model for human HBV infection and evaluation of Anti-HBV therapeutic agents. Hepatology (1999) 29(2):553–62. 10.1002/hep.510290228 9918935

[78] Ilan E Arazi J Nussbaum O Zauberman A Eren R Lubin I The hepatitis C virus (HCV)-Trimera mouse: a model for evaluation of agents against HCV. J Infect Dis (2002) 185(2):153–61. 10.1086/338266 11807688

[79] Meuleman P Leroux-Roels G. The human liver-uPA-SCID mouse: a model for the evaluation of antiviral compounds against HBV and HCV. Antiviral Res (2008) 80(3):231–8. 10.1016/j.antiviral.2008.07.006 18706933

[80] Eren R Landstein D Terkieltaub D Nussbaum O Zauberman A Ben-Porath J Preclinical evaluation of two neutralizing human monoclonal antibodies against hepatitis C virus (HCV): a potential treatment to prevent HCV reinfection in liver transplant patients. J Virol (2006) 80(6):2654–64. 10.1128/JVI.80.6.2654-2664.2006 16501075 PMC1395448

[81] Heckel JL Sandgren EP Degen JL Palmiter RD Brinster RL. Neonatal bleeding in transgenic mice expressing urokinase-type plasminogen activator. Cell (1990) 62(3):447–56. 10.1016/0092-8674(90)90010-C 1696178

[82] Sandgren EP Palmiter RD Heckel JL Daugherty CC Brinster RL Degen JL. Complete hepatic regeneration after somatic deletion of an albumin-plasminogen activator transgene. Cell (1991) 66(2):245–56. 10.1016/0092-8674(91)90615-6 1713128

[83] Mercer DF Schiller DE Elliott JF Douglas DN Hao C Rinfret A Hepatitis C virus replication in mice with chimeric human livers. Nat Med (2001) 7(8):927–33. 10.1038/90968 11479625

[84] Rhim JA Sandgren EP Degen JL Palmiter RD Brinster RL. Replacement of diseased mouse liver by hepatic cell transplantation. Science (1994) 263(5150):1149–52. 10.1126/science.8108734 8108734

[85] Petersen J Dandri M Gupta S Rogler CE. Liver repopulation with xenogenic hepatocytes in B and T cell-deficient mice leads to chronic hepadnavirus infection and clonal growth of hepatocellular carcinoma. Proc Natl Acad Sci U S A (1998) 95(1):310–5. 10.1073/pnas.95.1.310 9419372 PMC18210

[86] Rhim JA Sandgren EP Palmiter RD Brinster RL. Complete reconstitution of mouse liver with xenogeneic hepatocytes. Proc Natl Acad Sci U S A (1995) 92(11):4942–6. 10.1073/pnas.92.11.4942 7761429 PMC41823

[87] Dandri M Burda MR Torok E Pollok JM Iwanska A Sommer G Repopulation of mouse liver with human hepatocytes and in vivo infection with hepatitis B virus. Hepatology (2001) 33(4):981–8. 10.1053/jhep.2001.23314 11283864

[88] Tateno C Yoshizane Y Saito N Kataoka M Utoh R Yamasaki C Near completely humanized liver in mice shows human-type metabolic responses to drugs. Am J Pathol (2004) 165(3):901–12. 10.1016/S0002-9440(10)63352-4 15331414 PMC1618591

[89] Katoh M Sawada T Soeno Y Nakajima M Tateno C Yoshizato K In vivo drug metabolism model for human cytochrome P450 enzyme using chimeric mice with humanized liver. J Pharm Sci (2007) 96(2):428–37. 10.1002/jps.20783 17051594

[90] Meuleman P Libbrecht L De Vos R de Hemptinne B Gevaert K Vandekerckhove J Morphological and biochemical characterization of a human liver in a uPA-SCID mouse chimera. Hepatology (2005) 41(4):847–56. 10.1002/hep.20657 15791625

[91] Vanwolleghem T Libbrecht L Hansen BE Desombere I Roskams T Meuleman P Factors determining successful engraftment of hepatocytes and susceptibility to hepatitis B and C virus infection in uPA-SCID mice. J Hepatol (2010) 53(3):468–76. 10.1016/j.jhep.2010.03.024 20591528

[92] Bukh J Meuleman P Tellier R Engle RE Feinstone SM Eder G Challenge pools of hepatitis C virus genotypes 1-6 prototype strains: replication fitness and pathogenicity in chimpanzees and human liver-chimeric mouse models. J Infect Dis (2010) 201(9):1381–9. 10.1086/651579 20353362 PMC2941994

[93] Tateno C Kawase Y Tobita Y Hamamura S Ohshita H Yokomichi H Generation of novel chimeric mice with humanized livers by using hemizygous cDNA-uPA/SCID mice. PLoS One (2015) 10(11):e0142145. 10.1371/journal.pone.0142145 26536627 PMC4633119

[94] Suemizu H Hasegawa M Kawai K Taniguchi K Monnai M Wakui M Establishment of a humanized model of liver using NOD/Shi-scid IL2Rgnull mice. Biochem Biophys Res Commun (2008) 377(1):248–52. 10.1016/j.bbrc.2008.09.124 18840406

[95] Azuma H Paulk N Ranade A Dorrell C Al-Dhalimy M Ellis E Robust expansion of human hepatocytes in Fah(-/-)/Rag2(-/-)/Il2rg(-/-) mice. Nat Biotechnol (2007) 25(8):903–10. 10.1038/nbt1326 17664939 PMC3404624

[96] Bissig KD Wieland SF Tran P Isogawa M Le TT Chisari FV Human liver chimeric mice provide a model for hepatitis B and C virus infection and treatment. J Clin Invest (2010) 120(3):924–30. 10.1172/JCI40094 20179355 PMC2827952

[97] Uchida T Imamura M Kan H Hiraga N Hayes CN Tsuge M Usefulness of humanized cDNA-uPA/SCID mice for the study of hepatitis B virus and hepatitis C virus virology. J Gen Virol (2017) 98(5):1040–7. 10.1099/jgv.0.000726 28141486

[98] Weglarz TC Degen JL Sandgren EP. Hepatocyte transplantation into diseased mouse liver. Kinetics of parenchymal repopulation and identification of the proliferative capacity of tetraploid and octaploid hepatocytes. Am J Pathol (2000) 157(6):1963–74. 10.1016/S0002-9440(10)64835-3 11106569 PMC1885759

[99] Heo J Factor VM Uren T Takahama Y Lee JS Major M Hepatic precursors derived from murine embryonic stem cells contribute to regeneration of injured liver. Hepatology (2006) 44(6):1478–86. 10.1002/hep.21441 17133486

[100] Tesfaye A Stift J Maric D Cui Q Dienes HP Feinstone SM. Chimeric mouse model for the infection of hepatitis B and C viruses. PLoS One (2013) 8(10):e77298. 10.1371/journal.pone.0077298 24155939 PMC3796464

[101] Carpentier A Tesfaye A Chu V Nimgaonkar I Zhang F Lee SB Engrafted human stem cell-derived hepatocytes establish an infectious HCV murine model. J Clin Invest (2014) 124(11):4953–64. 10.1172/JCI75456 25295540 PMC4347235

[102] Wang Z Wu N Tesfaye A Feinstone S Kumar A. HCV infection-associated hepatocellular carcinoma in humanized mice. Infect Agent Cancer (2015) 10:24. 10.1186/s13027-015-0018-9 26217396 PMC4515941

[103] Hasegawa M Kawai K Mitsui T Taniguchi K Monnai M Wakui M The reconstituted ‘humanized liver’ in TK-NOG mice is mature and functional. Biochem Biophys Res Commun (2011) 405(3):405–10. 10.1016/j.bbrc.2011.01.042 21238430 PMC3648850

[104] Kosaka K Hiraga N Imamura M Yoshimi S Murakami E Nakahara T A novel TK-NOG based humanized mouse model for the study of HBV and HCV infections. Biochem Biophys Res Commun (2013) 441(1):230–5. 10.1016/j.bbrc.2013.10.040 24140055

[105] Uchida T Hiraga N Imamura M Yoshimi S Kan H Miyaki E Elimination of HCV via a non-ISG-mediated mechanism by vaniprevir and BMS-788329 combination therapy in human hepatocyte chimeric mice. Virus Res (2016) 213:62–8. 10.1016/j.virusres.2015.11.010 26569595

[106] Meuleman P Hesselgesser J Paulson M Vanwolleghem T Desombere I Reiser H Anti-CD81 antibodies can prevent a hepatitis C virus infection in vivo. Hepatology (2008) 48(6):1761–8. 10.1002/hep.22547 19030166

[107] Vanwolleghem T Bukh J Meuleman P Desombere I Meunier JC Alter H Polyclonal immunoglobulins from a chronic hepatitis C virus patient protect human liver-chimeric mice from infection with a homologous hepatitis C virus strain. Hepatology (2008) 47(6):1846–55. 10.1002/hep.22244 18452146

[108] Meuleman P Bukh J Verhoye L Farhoudi A Vanwolleghem T Wang RY In vivo evaluation of the cross-genotype neutralizing activity of polyclonal antibodies against hepatitis C virus. Hepatology (2011) 53(3):755–62. 10.1002/hep.24171 21319203 PMC3079546

[109] Meuleman P Catanese MT Verhoye L Desombere I Farhoudi A Jones CT A human monoclonal antibody targeting scavenger receptor class B type I precludes hepatitis C virus infection and viral spread in vitro and in vivo. Hepatology (2012) 55(2):364–72. 10.1002/hep.24692 21953761 PMC3262867

[110] Kneteman NM Weiner AJ O’Connell J Collett M Gao T Aukerman L Anti-HCV therapies in chimeric scid-Alb/uPA mice parallel outcomes in human clinical application. Hepatology (2006) 43(6):1346–53. 10.1002/hep.21209 16729319

[111] Joyce MA Walters KA Lamb SE Yeh MM Zhu LF Kneteman N HCV induces oxidative and ER stress, and sensitizes infected cells to apoptosis in SCID/Alb-uPA mice. PLoS Pathog (2009) 5(2):e1000291. 10.1371/journal.ppat.1000291 19242562 PMC2647842

[112] Lootens L Van Eenoo P Meuleman P Leroux-Roels G Delbeke FT. The uPA(+/+)-SCID mouse with humanized liver as a model for in vivo metabolism of 4-androstene-3,17-dione. Drug Metab Dispos (2009) 37(12):2367–74. 10.1124/dmd.109.028183 19741039

[113] Prentoe J Verhoye L Velazquez Moctezuma R Buysschaert C Farhoudi A Wang R HVR1-mediated antibody evasion of highly infectious in vivo adapted HCV in humanised mice. Gut (2016) 65(12):1988–97. 10.1136/gutjnl-2015-310300 26589670 PMC5136728

[114] Desombere I Mesalam AA Urbanowicz RA Van Houtte F Verhoye L Keck ZY A novel neutralizing human monoclonal antibody broadly abrogates hepatitis C virus infection in vitro and in vivo. Antiviral Res (2017) 148:53–64. 10.1016/j.antiviral.2017.10.015 29074219 PMC5785094

[115] Mailly L Xiao F Lupberger J Wilson GK Aubert P Duong FHT Clearance of persistent hepatitis C virus infection in humanized mice using a claudin-1-targeting monoclonal antibody. Nat Biotechnol (2015) 33(5):549–54. 10.1038/nbt.3179 25798937 PMC4430301

[116] Vercauteren K Van Den Eede N Mesalam AA Belouzard S Catanese MT Bankwitz D Successful anti-scavenger receptor class B type I (SR-BI) monoclonal antibody therapy in humanized mice after challenge with HCV variants with in vitro resistance to SR-BI-targeting agents. Hepatology (2014) 60(5):1508–18. 10.1002/hep.27196 24797654 PMC4211977

[117] Lacek K Vercauteren K Grzyb K Naddeo M Verhoye L Slowikowski MP Novel human SR-BI antibodies prevent infection and dissemination of HCV in vitro and in humanized mice. J Hepatol (2012) 57(1):17–23. 10.1016/j.jhep.2012.02.018 22414763

[118] Mesalam AA Vercauteren K Meuleman P. Mouse systems to model hepatitis C virus treatment and associated resistance. Viruses (2016) 8(6):E176. 10.3390/v8060176 27338446 PMC4926196

[119] Vercauteren K Brown RJ Mesalam AA Doerrbecker J Bhuju S Geffers R Targeting a host-cell entry factor barricades antiviral-resistant HCV variants from on-therapy breakthrough in human-liver mice. Gut (2016) 65(12):2029–34. 10.1136/gutjnl-2014-309045 26306759

[120] Khera T Todt D Vercauteren K McClure CP Verhoye L Farhoudi A Tracking HCV protease population diversity during transmission and susceptibility of founder populations to antiviral therapy. Antiviral Res (2017) 139:129–37. 10.1016/j.antiviral.2017.01.001 28062191 PMC5292934

[121] Sacci JB Jr Alam U Douglas D Lewis J Tyrrell DL Azad AF *Plasmodium falciparum* infection and exoerythrocytic development in mice with chimeric human livers. Int J Parasitol (2006) 36(3):353–60. 10.1016/j.ijpara.2005.10.014 16442544

[122] Foquet L Hermsen CC Verhoye L van Gemert GJ Cortese R Nicosia A Anti-CD81 but not anti-SR-BI blocks *Plasmodium falciparum* liver infection in a humanized mouse model. J Antimicrob Chemother (2015) 70(6):1784–7. 10.1093/jac/dkv019 25656410

[123] Foquet L Hermsen CC van Gemert GJ Van Braeckel E Weening KE Sauerwein R Vaccine-induced monoclonal antibodies targeting circumsporozoite protein prevent *Plasmodium falciparum* infection. J Clin Invest (2014) 124(1):140–4. 10.1172/JCI70349 24292709 PMC3871238

[124] Sayed IM Verhoye L Cocquerel L Abravanel F Foquet L Montpellier C Study of hepatitis E virus infection of genotype 1 and 3 in mice with humanised liver. Gut (2017) 66(5):920–9. 10.1136/gutjnl-2015-311109 27006186

[125] Montpellier C Wychowski C Sayed IM Meunier JC Saliou JM Ankavay M Hepatitis E virus lifecycle and identification of 3 forms of the ORF2 capsid protein. Gastroenterology (2018) 154(1):211–23.e8. 10.1053/j.gastro.2017.09.020 28958858

[126] Vercauteren K de Jong YP Meuleman P. HCV animal models and liver disease. J Hepatol (2014) 61(1 Suppl):S26–33. 10.1016/j.jhep.2014.07.013 25443343

[127] Grompe M al-Dhalimy M Finegold M Ou CN Burlingame T Kennaway NG Loss of fumarylacetoacetate hydrolase is responsible for the neonatal hepatic dysfunction phenotype of lethal albino mice. Genes Dev (1993) 7(12a):2298–307. 10.1101/gad.7.12a.2298 8253378

[128] Overturf K Al-Dhalimy M Tanguay R Brantly M Ou CN Finegold M Hepatocytes corrected by gene therapy are selected in vivo in a murine model of hereditary tyrosinaemia type I. Nat Genet (1996) 12(3):266–73. 10.1038/ng0396-266 8589717

[129] Grompe M Lindstedt S al-Dhalimy M Kennaway NG Papaconstantinou J Torres-Ramos CA Pharmacological correction of neonatal lethal hepatic dysfunction in a murine model of hereditary tyrosinaemia type I. Nat Genet (1995) 10(4):453–60. 10.1038/ng0895-453 7545495

[130] Bissig KD Le TT Woods NB Verma IM. Repopulation of adult and neonatal mice with human hepatocytes: a chimeric animal model. Proc Natl Acad Sci U S A (2007) 104(51):20507–11. 10.1073/pnas.0710528105 18077355 PMC2154461

[131] Lieber A Vrancken Peeters MJ Meuse L Fausto N Perkins J Kay MA. Adenovirus-mediated urokinase gene transfer induces liver regeneration and allows for efficient retrovirus transduction of hepatocytes in vivo. Proc Natl Acad Sci U S A (1995) 92(13):6210–4. 10.1073/pnas.92.13.6210 7597103 PMC41672

[132] Ellis EC Naugler WE Parini P Mork LM Jorns C Zemack H Mice with chimeric livers are an improved model for human lipoprotein metabolism. PLoS One (2013) 8(11):e78550. 10.1371/journal.pone.0078550 24223822 PMC3817217

[133] Calattini S Fusil F Mancip J Dao Thi VL Granier C Gadot N Functional and biochemical characterization of hepatitis C virus (HCV) particles produced in a humanized liver mouse model. J Biol Chem (2015) 290(38):23173–87. 10.1074/jbc.M115.662999 26224633 PMC4645586

[134] Burchill MA Roby JA Crochet N Wind-Rotolo M Stone AE Edwards MG Rapid reversal of innate immune dysregulation in blood of patients and livers of humanized mice with HCV following DAA therapy. PLoS One (2017) 12(10):e0186213. 10.1371/journal.pone.0186213 29040318 PMC5645093

[135] Sayed IM Foquet L Verhoye L Abravanel F Farhoudi A Leroux-Roels G Transmission of hepatitis E virus infection to human-liver chimeric FRG mice using patient plasma. Antiviral Res (2017) 141:150–4. 10.1016/j.antiviral.2017.02.011 28232247

[136] de Jong YP Dorner M Mommersteeg MC Xiao JW Balazs AB Robbins JB Broadly neutralizing antibodies abrogate established hepatitis C virus infection. Sci Trans Med (2014) 6(254):254ra129 10.1126/scitranslmed.3009512 PMC4312107 25232181

[137] Andreo U de Jong YP Scull MA Xiao JW Vercauteren K Quirk C Analysis of hepatitis C virus particle heterogeneity in immunodeficient human liver chimeric fah-/- mice. Cell Mol Gastroenterol Hepatol (2017) 4(3):405–17. 10.1016/j.jcmgh.2017.07.002 28936471 PMC5602752

[138] Flannery EL Foquet L Chuenchob V Fishbaugher M Billman Z Navarro MJ Assessing drug efficacy against *Plasmodium falciparum* liver stages in vivo. JCI Insight (2018) 3(1):92587. 10.1172/jci.insight.92587 29321371 PMC5821200

[139] Ouyang EC Wu CH Walton C Promrat K Wu GY. Transplantation of human hepatocytes into tolerized genetically immunocompetent rats. World J Gastroenterol (2001) 7(3):324–30. 10.3748/wjg.v7.i3.324 11819784 PMC4688716

[140] Kline GM Shen Z Mohiuddin M Ruggiero V Rostami S DiSesa VJ. Development of tolerance to experimental cardiac allografts in utero. Ann Thorac Surg (1994) 57(1):72–4. 10.1016/0003-4975(94)90367-0 8279922

[141] Wu GY Konishi M Walton CM Olive D Hayashi K Wu CH. A novel immunocompetent rat model of HCV infection and hepatitis. Gastroenterology (2005) 128(5):1416–23. 10.1053/j.gastro.2005.03.015 15887122

[142] Washburn ML Bility MT Zhang L Kovalev GI Buntzman A Frelinger JA A humanized mouse model to study hepatitis C virus infection, immune response, and liver disease. Gastroenterology (2011) 140(4):1334–44. 10.1053/j.gastro.2011.01.001 21237170 PMC3066273

[143] Pajvani UB Trujillo ME Combs TP Iyengar P Jelicks L Roth KA Fat apoptosis through targeted activation of caspase 8: a new mouse model of inducible and reversible lipoatrophy. Nat Med (2005) 11(7):797–803. 10.1038/nm1262 15965483

[144] Traggiai E Chicha L Mazzucchelli L Bronz L Piffaretti JC Lanzavecchia A Development of a human adaptive immune system in cord blood cell-transplanted mice. Science (2004) 304(5667):104–7. 10.1126/science.1093933 15064419

[145] Bility MT Nio K Li F McGivern DR Lemon SM Feeney ER Chronic hepatitis C infection-induced liver fibrogenesis is associated with M2 macrophage activation. Sci Rep (2016) 6:39520. 10.1038/srep39520 28000758 PMC5175173

[146] Douglas DN Kneteman NM. Generation of improved mouse models for the study of hepatitis C virus. Eur J Pharmacol (2015) 759:313–25. 10.1016/j.ejphar.2015.03.022 25814250

[147] Gutti TL Knibbe JS Makarov E Zhang J Yannam GR Gorantla S Human hepatocytes and hematolymphoid dual reconstitution in treosulfan-conditioned uPA-NOG mice. Am J Pathol (2014) 184(1):101–9. 10.1016/j.ajpath.2013.09.008 24200850 PMC3873481

[148] Wilson EM Bial J Tarlow B Bial G Jensen B Greiner DL Extensive double humanization of both liver and hematopoiesis in FRGN mice. Stem Cell Res (2014) 13(3 Pt A):404–12. 10.1016/j.scr.2014.08.006 25310256 PMC7275629

[149] Strick-Marchand H Dusseaux M Darche S Huntington ND Legrand N Masse-Ranson G A novel mouse model for stable engraftment of a human immune system and human hepatocytes. PLoS One (2015) 10(3):e0119820. 10.1371/journal.pone.0119820 25782010 PMC4364106

[150] Dusseaux M Masse-Ranson G Darche S Ahodantin J Li Y Fiquet O Viral load affects the immune response to HBV in mice with humanized immune system and liver. Gastroenterology (2017) 153(6):1647–61.e9. 10.1053/j.gastro.2017.08.034 28851562 PMC5733397

[151] Bility MT Cheng L Zhang Z Luan Y Li F Chi L Hepatitis B virus infection and immunopathogenesis in a humanized mouse model: induction of human-specific liver fibrosis and M2-like macrophages. PLoS Pathog (2014) 10(3):e1004032 10.1371/journal.ppat.1004032 24651854 PMC3961374

[152] Shultz LD Saito Y Najima Y Tanaka S Ochi T Tomizawa M Generation of functional human T-cell subsets with HLA-restricted immune responses in HLA class I expressing NOD/SCID/IL2r gamma(null) humanized mice. Proc Natl Acad Sci U S A (2010) 107(29):13022–7. 10.1073/pnas.1000475107 20615947 PMC2919921

[153] Mignon A Guidotti JE Mitchell C Fabre M Wernet A De La Coste A Selective repopulation of normal mouse liver by Fas/CD95-resistant hepatocytes. Nat Med (1998) 4(10):1185–8. 10.1038/2681 9771754

[154] Chen Q Khoury M Limmon G Choolani M Chan JK Chen J. Human fetal hepatic progenitor cells are distinct from, but closely related to, hematopoietic stem/progenitor cells. Stem Cells (2013) 31(6):1160–9. 10.1002/stem.1359 23404852

[155] Keng CT Sze CW Zheng D Zheng Z Yong KS Tan SQ Characterisation of liver pathogenesis, human immune responses and drug testing in a humanised mouse model of HCV infection. Gut (2016) 65(10):1744–53. 10.1136/gutjnl-2014-307856 26149491 PMC5036242

[156] Zheng Z Sze CW Keng CT Al-Haddawi M Liu M Tan SY Hepatitis C virus mediated chronic inflammation and tumorigenesis in the humanised immune system and liver mouse model. PLoS One (2017) 12(9):e0184127. 10.1371/journal.pone.0184127 28886065 PMC5590885

[157] Bitzegeio J Bankwitz D Hueging K Haid S Brohm C Zeisel MB Adaptation of hepatitis C virus to mouse CD81 permits infection of mouse cells in the absence of human entry factors. PLoS Pathog (2010) 6(7):e1000978. 10.1371/journal.ppat.1000978 20617177 PMC2895659

[158] von Schaewen M Dorner M Hueging K Foquet L Gerges S Hrebikova G Expanding the host range of hepatitis C virus through viral adaptation. MBio (2016) 7(6):e1915–6. 10.1128/mBio.01915-16 27834208 PMC5101358

[159] Dorner M Horwitz JA Donovan BM Labitt RN Budell WC Friling T Completion of the entire hepatitis C virus life cycle in genetically humanized mice. Nature (2013) 501(7466):237. 10.1038/nature12427 23903655 PMC3858853

[160] Ding Q Schaewen M Hrebikova G Heller B Sandmann L Plaas M Mice expressing minimally humanized CD81 and occludin genes support hepatitis C virus uptake in vivo. J Virol (2017) 91(4):e1799–1716. 10.1128/JVI.01799-16 27928007 PMC5286898

[161] Chen J Zhao Y Zhang C Chen H Feng J Chi X Persistent hepatitis C virus infections and hepatopathological manifestations in immune-competent humanized mice. Cell Res (2014) 24(9):1050–66. 10.1038/cr.2014.116 25155355 PMC4152738

[162] Long G Hiet MS Windisch MP Lee JY Lohmann V Bartenschlager R. Mouse hepatic cells support assembly of infectious hepatitis C virus particles. Gastroenterology (2011) 141(3):1057–66. 10.1053/j.gastro.2011.06.010 21699799

[163] Stapleton JT Foung S Muerhoff AS Bukh J Simmonds P. The GB viruses: a review and proposed classification of GBV-A, GBV-C (HGV), and GBV-D in genus *Pegivirus* within the family *Flaviviridae*. J Gen Virol (2011) 92(Pt 2):233–46. 10.1099/vir.0.027490-0 21084497 PMC3081076

[164] Deinhardt F Holmes AW Capps RB Popper H Studies on the transmission of human viral hepatitis to marmoset monkeys. J Exp Med (1967) 125(4):673–88. 10.1084/jem.125.4.673 4960092 PMC2138365

[165] Bukh J Engle RE Govindarajan S Purcell RH. Immunity against the GBV-B hepatitis virus in tamarins can prevent productive infection following rechallenge and is long-lived. J Med Virol (2008) 80(1):87–94. 10.1002/jmv.21013 18041000

[166] Bright H Carroll AR Watts PA Fenton RJ. Development of a GB virus B marmoset model and its validation with a novel series of hepatitis C virus NS3 protease inhibitors. J Virol (2004) 78(4):2062–71. 10.1128/JVI.78.4.2062-2071.2004 14747571 PMC369465

[167] Takikawa S Engle RE Faulk KN Emerson SU Purcell RH Bukh J. Molecular evolution of GB virus B hepatitis virus during acute resolving and persistent infections in experimentally infected tamarins. J Gen Virol (2010) 91(Pt 3):727–33. 10.1099/vir.0.015750-0 19906942 PMC2888096

[168] Scheel TKH Simmonds P Kapoor A. Surveying the global virome: identification and characterization of HCV-related animal hepaciviruses. Antiviral Res (2015) 115:83–93. 10.1016/j.antiviral.2014.12.014 25545071 PMC5081135

[169] Kapoor A Simmonds P Gerold G Qaisar N Jain K Henriquez JA Characterization of a canine homolog of hepatitis C virus. Proc Natl Acad Sci U S A (2011) 108(28):11608–13. 10.1073/pnas.1101794108 21610165 PMC3136326

[170] Kapoor A Simmonds P Scheel TKH Hjelle B Cullen JM Burbelo PD Identification of rodent homologs of hepatitis C virus and pegiviruses. MBio (2013) 4(2):e00216–13. 10.1128/mBio.00216-13 23572554 PMC3622934

[171] Drexler JF Corman VM Müller MA Lukashev AN Gmyl A Coutard B Evidence for novel hepaciviruses in rodents. PLoS Pathog (2013) 9(6):e1003438. 10.1371/journal.ppat.1003438 23818848 PMC3688547

[172] Firth C Bhat M Firth MA Williams SH Frye MJ Simmonds P Detection of zoonotic pathogens and characterization of novel viruses carried by commensal *Rattus norvegicus* in New York City. MBio (2014) 5(5):e01933–14. 10.1128/mBio.01933-14 25316698 PMC4205793

[173] Billerbeck E Wolfisberg R Fahnøe U Xiao JW Quirk C Luna JM Mouse models of acute and chronic hepacivirus infection. Science (2017) 357(6347):204–8. 10.1126/science.aal1962 28706073 PMC5654634

[174] Trivedi S Murthy S Sharma H Hartlage AS Kumar A Gadi S Viral persistence, liver disease and host response in hepatitis C-like virus rat model. Hepatology (2017). 10.1002/hep.29494 28859226 PMC5832584

